# Personalised, Rational, Efficacy-Driven Cancer Drug Dosing *via* an Artificial Intelligence SystEm (PRECISE): A Protocol for the PRECISE CURATE.AI Pilot Clinical Trial

**DOI:** 10.3389/fdgth.2021.635524

**Published:** 2021-04-12

**Authors:** Benjamin Kye Jyn Tan, Chong Boon Teo, Xavier Tadeo, Siyu Peng, Hazel Pei Lin Soh, Sherry De Xuan Du, Vilianty Wen Ya Luo, Aishwarya Bandla, Raghav Sundar, Dean Ho, Theodore Wonpeum Kee, Agata Blasiak

**Affiliations:** ^1^Yong Loo Lin School of Medicine, National University of Singapore, Singapore, Singapore; ^2^The N.1 Institute for Health (N.1), National University of Singapore, Singapore, Singapore; ^3^The Institute for Digital Medicine (WisDM), Yong Loo Lin School of Medicine, National University of Singapore, Singapore, Singapore; ^4^Department of Biomedical Engineering, NUS Engineering, National University of Singapore, Singapore, Singapore; ^5^Department of Medicine, National University Health System, Singapore, Singapore; ^6^Haematology-Oncology Research Group, National University Cancer Institute, Singapore (NCIS), Singapore, Singapore; ^7^Department of Haematology-Oncology, National University Health System, Singapore, Singapore; ^8^Department of Pharmacology, Yong Loo Lin School of Medicine, National University of Singapore, Singapore, Singapore; ^9^Smart Systems Institute, National University of Singapore, Singapore, Singapore

**Keywords:** clinical decision support system, chemotherapy, personalised medicine, clinical trials, artificial intelligence, oncology, precision medicine, PRECISE CURATE.AI pilot clinical trial

## Abstract

**Introduction:** Oncologists have traditionally administered the maximum tolerated doses of drugs in chemotherapy. However, these toxicity-guided doses may lead to suboptimal efficacy. CURATE.AI is an indication-agnostic, mechanism-independent and efficacy-driven personalised dosing platform that may offer a more optimal solution. While CURATE.AI has already been applied in a variety of clinical settings, there are no prior randomised controlled trials (RCTs) on CURATE.AI-guided chemotherapy dosing for solid tumours. Therefore, we aim to assess the technical and logistical feasibility of a future RCT for CURATE.AI-guided solid tumour chemotherapy dosing. We will also collect exploratory data on efficacy and toxicity, which will inform RCT power calculations.

**Methods and analysis:** This is an open-label, single-arm, two-centre, prospective pilot clinical trial, recruiting adults with metastatic solid tumours and raised baseline tumour marker levels who are planned for palliative-intent, capecitabine-based chemotherapy. As CURATE.AI is a small data platform, it will guide drug dosing for each participant based only on their own tumour marker levels and drug doses as input data. The primary outcome is the proportion of participants in whom CURATE.AI is successfully applied to provide efficacy-driven personalised dosing, as judged based on predefined considerations. Secondary outcomes include the timeliness of dose recommendations, participant and physician adherence to CURATE.AI-recommended doses, and the proportion of clinically significant dose changes. We aim to initially enrol 10 participants from two hospitals in Singapore, perform an interim analysis, and consider either cohort expansion or an RCT. Recruitment began in August 2020. This pilot clinical trial will provide key data for a future RCT of CURATE.AI-guided personalised dosing for precision oncology.

**Ethics and dissemination:** The National Healthcare Group (NHG) Domain Specific Review Board has granted ethical approval for this study (DSRB 2020/00334). We will distribute our findings at scientific conferences and publish them in peer-reviewed journals.

**Trial registration number:** NCT04522284

## Introduction

Treatment options for oncology patients are increasingly personalised with the onset of precision medicine, which enables drug selection tailored to an individual. To truly optimise the outcome, however, the treatment should also include personalised dosing (1). Faced with a trade-off between efficacy and toxicity, oncologists traditionally administer maximum tolerated dose (MTD)—the highest dose that does not cause unacceptable side effects (2), derived from Phase I trials. If patients experience toxicities, the dose is reduced according to guidelines based on population data. The key underlying assumption is that higher doses will provide greater efficacy, which many now recognize as flawed (3). Large randomised controlled trials (RCTs) show that doubling the dose of some drugs results in more frequent toxicities, which leads to dose reductions and treatment interruptions, despite no improvement in overall survival (4, 5). Conversely, other authors suggest that 30–75% of patients may be underdosed with conventional dosing strategies (6). Given the narrow therapeutic window of oncologic drugs and the 4- to 10-fold interindividual variation in pharmacokinetic clearance (7, 8), these overdosing or underdosing events result in suboptimal results: unnecessary toxicities, low efficacy and potential failure of drug development (9). The complexity of this problem increases exponentially in multi-drug regimens, with unpredictable drug-drug interactions (10, 11).

The challenge of personalised dosing has thus far been practically unsolvable because of its monumental complexity. Pharmacokinetic-based therapeutic drug monitoring and pharmacogenetic testing are promising (12–14), but have thus far not been adopted in chemotherapy drug dosing. In addition, threshold drug exposure does not necessarily correlate with threshold efficacy, particularly due to the individualised nature of patient responses to treatment. With the advent of artificial intelligence (AI), efforts are now focused on big data in pharmacogenomics (15). Though big data modelling is valuable, it is unsuitable for dynamic, multifactorial diseases, as it requires massive volumes of population information, comprehensive prior knowledge and high temporal resolution (16).

The alternative is to use small data, where only the individual's medical profile is used solely for his/her own treatment. Harnessing AI, we previously demonstrated that a quadratic surface can closely describe the relationship between drug doses and a phenotypic response in a human system (17–21). The coefficients of the second-order polynomial are unique to each individual. AI is used to map the extensive dose-response parameter space based on minimal empirical data, thus deterministically predicting the global optimum within a pre-specified safe dose range (22). Uniquely, this process is mechanism-independent and implicitly avoids the complexities stemming from the known or unknown mechanistic components, including pharmacokinetics, pharmacodynamics, pharmacogenomics, and disease biology (23, 24). These personalised profiles, which are visually represented as response surfaces, can also be scaled into higher dimensions to enable predictions for combination drug regimens (25). As such, CURATE.AI—a small data, AI-derived, indication-agnostic and mechanism-independent technology platform—could thus allow for clinically actionable personalised dosing for precision oncology and beyond. Importantly, CURATE.AI implementation is not purely computational, as it pairs prospective calibration of patient-specific responses to treatment at different drug doses with the dose optimisation process.

Predicting the optimal dose at a single point in time is only part of the challenge. The optimal dose evolves throughout the course of treatment, with the participant's response to therapy evolving due to environmental, physiologic and disease changes, including drug resistance (7). Here, CURATE.AI's ability to continually recalibrate personalised profiles allows for dynamic dose optimisation throughout treatment (23, 24). To maximise CURATE.AI's therapeutic potential, it is thus paramount to utilize a phenotypic response biomarker that can be quantified frequently. Though the gold standard for monitoring treatment response in solid tumours is the Response Evaluation Criteria in Solid Tumours (RECIST), these radiological assessments are performed at infrequent intervals after a few chemotherapy cycles, and may not reflect the entire tumour burden in situations such as widespread metastases or central necrosis (26). Blood-based tumour markers, such as carcinoembryonic antigen (CEA) and carbohydrate antigen 19-9 (CA19-9) (27–29), can be measured more frequently. However, these traditional blood biomarkers have limited sensitivity and specificity in reflecting tumour response, especially among patients whose baseline biomarker levels are in the normal range (30). Recent advances in genomic sequencing have revealed plasma circulating tumour DNA (ctDNA) as a novel biomarker of tumour burden (31). ctDNA are DNA fragments released by tumour cells, *via* apoptosis, necrosis or active secretion. These fragments contain both genetic information (such as point mutation, copy number variation) and epigenetic information (such as DNA methylation). Quantitative changes in ctDNA have been shown to be ultra-sensitive in detecting both macroscopic and microscopic tumour load (32), including in patients without raised traditional tumour markers at baseline. Therefore, serial ctDNA measurements may be more reflective of the evolving tumour response and thus appropriate for CURATE.AI.

Though CURATE.AI has already been applied in a variety of clinical settings (23–25), there are currently no prior RCTs of CURATE.AI in patients with solid tumours. Additionally, the temporal resolution of the change in the selected tumour markers, as required for CURATE.AI, has not been yet characterised. We are thus conducting this pilot clinical trial to assess the technical and logistical feasibility of a future RCT, as well as to collect exploratory data on efficacy and toxicity to enable future sample size calculations.

## Methods and Analysis

### Trial Design

The PRECISE CURATE.AI pilot trial is a single-arm, prospective pilot clinical trial for participants receiving palliative-intent chemotherapy for solid tumours. We anticipate that any feasibility concerns in the future RCT are unlikely to arise from the logistical process or participants acceptability of randomisation. Rather, the more pressing feasibility issues relate to whether CURATE.AI profiles can be created and successfully applied, and whether the recommended doses are different from the standard-of-care. Therefore, to maximise clinical experience in delivering this intervention, we will run this pilot as a single-arm study. This protocol adheres to the Consolidated Standards of Reporting Trials (CONSORT) extension for randomised pilot and feasibility trials (33).

### Study Setting and Participant Recruitment

We will recruit participants at two centres: National University Hospital (NUH) and Ng Teng Fong General Hospital (NTFGH), which both belong to the National University Health System (NUHS), Singapore. Clinical investigators will recruit patients from outpatient clinics during routine clinical reviews, weekly trial meetings or multidisciplinary tumour boards. As this is a pilot clinical trial with no precedent data, we intend to first recruit 10 patients and perform an interim analysis, which will include formal power and statistical sample size calculations. Based on these outcomes, we will consider an RCT, or an amendment of this study protocol to expand the current cohort or to include further cohorts of patients with a wider range of chemotherapy regimens or tumour markers. We will consider participants to be evaluable for the primary outcome measures only if participants are able to complete the first two cycles of chemotherapy in an uninterrupted manner. We will replace any unevaluable participants with new participants, though we will continue to monitor and report any adverse events in the unevaluable participants. We will consider replacing participants who withdraw from the study (see Supplementary Text) with new participants if we could not collect sufficient data for primary outcome assessment.

### Choice of Chemotherapy Regimens

Among the different solid tumour types, gastrointestinal (GI) tumours are one example of an appropriate solid tumour type for an initial trial with CURATE.AI. This is because they are the most prevalent (34), and they also form a significant proportion of tumours with raised levels of serum tumour markers (35), which can serve as an input for CURATE.AI. Therefore, we intend to initially recruit patients with GI tumours for this pilot clinical trial, though the eligibility criteria below permit the recruitment of patients with other solid tumour types.

We chose capecitabine-containing regimens (XELOX, XELIRI and single-agent capecitabine) as capecitabine is one of the most commonly used drugs for a wide range of GI and other solid tumours (36), which facilitates recruitment. In addition, it is generally accepted that the MTD for capecitabine is not well-tolerated in a large proportion of patients (37, 38), with guidelines recommending a starting dose lower than the MTD (39). Furthermore, previous studies have established inter-ethnic differences in tolerability profiles for capecitabine (40). Therefore, patients receiving capecitabine require a personalised approach and may benefit from CURATE.AI dose guidance.

During the trial, participants will receive the treatment in 3-week cycles, according to standard dosing schedules for each regimen (**Figure 2**) (41–43). In this pilot clinical trial, CURATE.AI will only modulate capecitabine doses while the remaining drugs (oxaliplatin in XELOX, and irinotecan in XELIRI regimens) will be held constant or adjusted at the clinical investigator's discretion, as per standard-of-care. Additionally, CURATE.AI will only modulate capecitabine doses between cycles and not within a cycle. The drugs used in these regimens will be subjected to the same storage and accountability conditions as per standard-of-care institutional requirements.

### Eligibility Criteria

The key inclusion criteria are as follows: (1) metastatic solid tumours not for curative intent therapy; (2) planned for treatment with the following chemotherapy regimens: XELOX, XELIRI or single-agent capecitabine; (3) presence of raised tumour marker above upper limit of local laboratory normal (e.g., CEA, CA19-9); (4) males and females ≥21 years of age (the age of majority in Singapore); (5) Eastern Cooperative Oncology Group (ECOG) Performance Status of 0 to 2; (6) meet the following clinical laboratory criteria within 21 days of starting treatment: (a) Absolute neutrophil count (ANC) ≥1,000/mm3 and platelet ≥50,000/mm3, (b) total bilirubin ≤ 1.5x the upper limit of the normal range (ULN) and alanine aminotransferase (ALT) and aspartate aminotransferase (AST) ≤ 3x ULN or ≤ 5x ULN in the of the liver involvement, (c) calculated creatinine clearance ≥30 mL/min or creatinine <1.5x ULN.

The key exclusion criteria as follows: (1) currently lactating or pregnant; (2) major surgery within 28 days prior to start of the treatment; (3) active congestive heart failure (New York Heart Association [NYHA] Class III or IV), symptomatic ischaemia, conduction abnormalities uncontrolled by conventional intervention or myocardial infarction within 4 months prior to date of obtaining informed consent; (4) clinically significant hypersensitivity to one or more of the selected regimen's constituent drug(s); (5) contraindication to any of the required concomitant drugs or supportive treatments; (6) any clinically significant medical disease or psychiatric condition that, in the investigator's opinion, may interfere with protocol adherence or a participant's ability to give informed consent.

### Interventions

#### Definition of CURATE.AI

**Figure 1:**
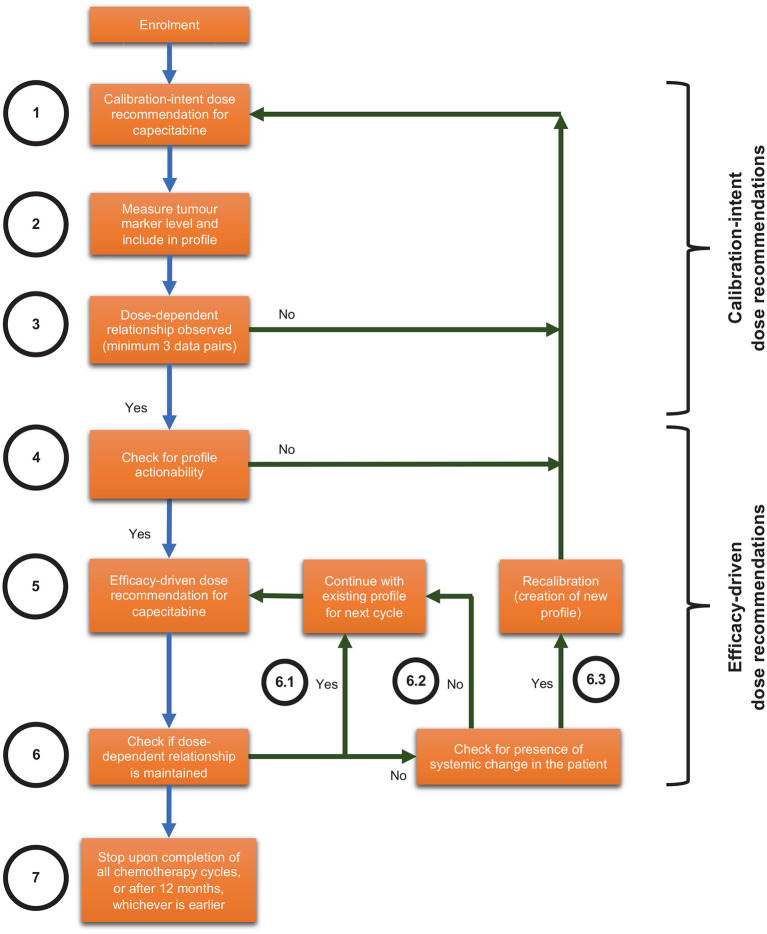
Internal workflow for optimising combination therapy modulation with CURATE.AI for solid tumours, including scenarios that may lead to recalibration.

CURATE.AI in this context refers to the CURATE.AI platform (software), engineering expertise in operating the CURATE.AI platform, drug dose recommendations generated by the CURATE.AI platform and accompanying analyses of clinical data. The Health Sciences Authority in Singapore classifies CURATE.AI as a Class B medical device (low to moderate risk), which is defined as all active therapeutic devices that are software, or which are intended to administer or exchange energy to or with the human body. We have filed the accompanying Clinical Research Materials notification under the National University of Singapore, for the intended purpose of providing dose recommendations within this pilot clinical trial. The CURATE.AI internal workflow is summarised in Figure 1 and explained in subsequent sections.

#### CURATE.AI Built-In Safety Mechanisms

All doses recommended by CURATE.AI will always be within the safe dose range pre-specified by the clinical investigator: (1) the predetermined safety range (50–100% of dose used in standard-of-care treatment) and (2) participant-specific dosing range (accounting for the specific participant's personal medical history and clinical context). If CURATE.AI is unable to recommend a dose that fulfils the above requirements, it will not recommend a modulated dose and the clinical investigator will decide the dose according to the standard-of-care. Should the participant experience clinically relevant grade 3 or 4 non-haematological toxicity at a particular dose, we will restrict the next dose recommendation by CURATE.AI to a lower dose. Clinical investigators will have the final say on whether to use the CURATE.AI-recommended dose. Clinical investigators may also adjust the dose beyond CURATE.AI's recommendations as they deem necessary.

#### CURATE.AI Calibration-Intent Dose Recommendations

For every participant, CURATE.AI will undergo an initial calibration stage (Figure 1, Steps 1–4) with the aim of generating a personalised CURATE.AI profile based on that participant data only. CURATE.AI will provide calibration-intent dose recommendations (Figure 1, Step 1) to collect data on the participant's phenotypic response (measured by tumour markers e.g., CEA, CA19-9) to a range of drug doses (Figure 1, Step 2). Any dose recommended by CURATE.AI to the clinical investigator will always be within the pre-specified safe dose range.

CURATE.AI requires a minimum of three unique data pairs (capecitabine dose and tumour marker), with one data pair collected per cycle, to generate the personalised CURATE.AI profile (Figure 1, Step 3) based on a second-order polynomial. After giving participants capecitabine at calibration-intent doses and measuring the corresponding tumour marker levels, we will analyse the data to determine if they fulfil calibration requirements. If not, CURATE.AI will make further calibration-intent dose recommendations and more data pairs will be collected until the CURATE.AI profile can be generated.

#### CURATE.AI Efficacy-Driven Dose Recommendations

After the CURATE.AI profile is generated, we will check it for actionability, defined as the ability to recommend an optimum dose within the safety requirements pre-specified by the clinical investigator (Figure 1, Step 4). For participants with actionable profiles, CURATE.AI will recommend therapeutic-intent, efficacy-driven doses to the clinical investigator (Figure 1, Step 5). Any dose recommended by CURATE.AI will always be within the safe dose range pre-specified by the clinical investigator. If the dose-dependent relationship is maintained, CURATE.AI will continue to use the existing profile to provide dynamic dose recommendations before every subsequent cycle (Figure 1, Step 6.1). This continues until the end of the participant's involvement in the study (Figure 1, Step 7), which is when the clinical investigator decides to cease/change the chemotherapy regimen, or until 12 months, whichever is earlier. If the dose-dependent relationship is not maintained but we do not suspect systemic changes in the participant (Figure 1, Step 6.2), CURATE.AI will obtain a new data pair in the next cycle based on the existing profile. If the dose-dependent relationship is not maintained and we suspect systemic changes in the participant at any point during the trial, such as but not limited to the introduction of haemodialysis or drugs with known interactions with the treatment, CURATE.AI may recalibrate the personalised profile (Figure 1, Step 6.3) to generate a new profile for the participant. CURATE.AI will select recalibration doses on the basis of previous correlations.

### Objectives

#### Primary Objective

Our primary objective is to assess the technical and logistical feasibility of an RCT for CURATE.AI-guided dosing with the selected chemotherapy regimens and tumour markers. Technical feasibility is defined by the following questions: (1) whether CURATE.AI profiles can be successfully created and applied; (2) whether the specific patient characteristics that predict the successful creation and applicable of CURATE.AI profiles can be identified; (3) whether the CURATE.AI-recommended dose is substantially different from the standard-of-care dose (see Table 1 footnote e). Logistical feasibility refers to: (1) the timeliness of CURATE.AI dose recommendations to the physician; (2) participant adherence to CURATE.AI-recommended doses; (3) physician adherence to CURATE.AI recommended doses. We hypothesize that CURATE.AI will meet these feasibility criteria, as further defined in our outcome measures below, for the selected palliative-intent chemotherapy regimens and respective tumour markers. We will use these data to decide if and how a future RCT should proceed.

**Table 1:** Outcome measures and progression criteria for the PRECISE CURATE.AI pilot clinical trial, based on “the traffic light system” recommended in the CONSORT extension statement for pilot clinical trials (33).

**Outcome measures**	Green^*^	Yellow^*^	Red^*^
**Primary Outcome Measures**			
Applicability of CURATE.AI profiles^a^	>70%	10–70%	<10%
**Secondary Outcome Measures**			
Patient adherence^b^	>90%	10–90%	<10%
Timeliness of CURATE.AI dose recommendations to the physician^c^	100%	10–99%	<10%
Physician adherence^d^	>70%	10–70%	<10%
Clinically significant dose changes^e^	>20%	1–20%	0%
**Exploratory Outcome Measures**			
Efficacy: (1) Clinical progressive disease^f^ (2) Temporal variation in tumour marker level (3) Maximal reduction in tumour marker level	N.A.	N.A.	N.A.
Clinically relevant toxicities^g^	N.A.	N.A.	N.A.
Data collection and exploratory analysis of CEA, CA19-9, and/or other traditional markers in higher frequency serial measurements after modulated dosing in relation to standard frequency readings and other efficacy measures (e.g., RECIST criteria).	N.A.	N.A.	N.A.
Data collection and exploratory analysis of ctDNA as: (1) a tumour marker in serial measurements at given clinical context and after modulated dosing (2) a potential input for CURATE.AI	N.A.	N.A.	N.A.

*See section on Data Collection, Management and Analysis.*

* *Green: a future randomised trial is definitely feasible. Yellow: a future randomised trial is possibly feasible if its design is appropriately modified. Red: a future randomised trial is unfeasible in its current form.*

a *percentage of participants in whom we successfully apply CURATE.AI profile. A decision on whether we “successfully apply” the CURATE.AI profile requires expert judgement and cannot be made based on a purely numerical process. The expert panel will consider the following factors with careful regard for the individual circumstances of each participant: (1) error/variance (biological/analytical) is allows accurate predictions (see section primary outcome measure); (2) profile can be generated sufficiently early for the participant to potentially benefit; (3) dose-dependent relationship is observed; (4) profile is actionable (i.e., fulfils the clinical investigator's pre-specified safety requirements); (5) systemic changes in the participant which require profile recalibration are rare or readily assimilated into the CURATE.AI algorithm.*

b *percentage of participants who always adhered to the prescribed dose whenever they took their medication, as measured by the standardised pharmacovigilance protocol.*

c *percentage of CURATE.AI recommendations provided in time for the next chemotherapy cycle, across all participants and cycles.*

d *percentage of CURATE.AI recommended doses that were used by the clinical investigator.*

e *percentage of participants in whom the CURATE.AI-guided cumulative dose is substantially (≥10%) different from the projected standard-of-care cumulative dose, which is defined as the maximum dose of capecitabine (1,000 mg/m^2^ twice daily for XELOX and XELIRI regimen, 1,250 mg/m^2^ twice daily for single agent capecitabine regimen) multiplied by the number of completed chemotherapy cycles.*

f *defined as the clinical investigator deeming that the patient will not benefit any further from the chemotherapy regimen and considering stopping it, at the time of the first radiological assessment performed as per standard-of-care.*

g *of grades 3–4 based on Common Terminology Criteria for Adverse Events (CTCAE) version 4.0.*

*ctDNA, circulating tumour DNA*.

#### Secondary Objective

Our secondary objective is to collect preliminary data on efficacy and toxicity as exploratory outcomes for this pilot. Specifically, we will evaluate the incidence of clinically progressive disease, changes in tumour marker levels, and incidence of clinically relevant toxicities. As an exploratory outcome, we will explore the utility of tumour markers such as CEA, CA19-9 and ctDNA in higher-frequency serial measurements with modulated doses. We will also explore ctDNA as an input for CURATE.AI to generate dose recommendations, however we will not use this analysis to prospectively guide dosing. We provide further details in our outcome measures below.

### Study Timeline and Investigations

**Figure 2:**
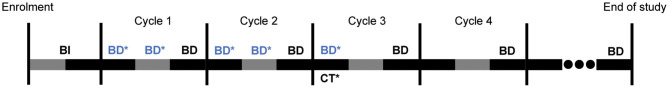
Overall trial schedule for 3 weeks long cycles. BI: Baseline investigations as per standard-of-care, including collection of demographics, medical/treatment history, vital signs, and conducting complete physical examination including performance status evaluation, blood tests [haematology, serum chemistries, tumour marker(s)] and imaging as clinically required. BD: Mandatory blood draws done every 3 weeks for the measurement of tumour marker(s) between Days 17–20 of each cycle, alongside serum chemistries and haematology as per standard-of-care. BD*: Additional blood draws for measurements of tumour markers(s), done once weekly (once between Days 4–7, and another between Days 11–14) performed solely for the purposes of the trial, and are necessary for cycles 1–2 of chemotherapy. Additional blood draws in subsequent cycles will be limited to one draw, if at all. CT*: Computed tomography scans performed approximately every two or three cycles as per standard-of-care. End of study: upon completion of all chemotherapy cycles, or at 12 months, whichever is earlier for each participant.

The study investigations schedule is summarised in Figure 2. For each participant, we will perform regular reviews of medical history, physical examination including performance status and vital signs, and documentation of adverse events and concomitant medications as per standard-of-care. We will also perform standard-of-care investigations at regular intervals, such as but not limited to: haematology, serum chemistries, traditional tumour markers (e.g., CEA or CA19-9) and computed tomography (CT) scans. We will conduct these investigations following standard-of-care institutional laboratory techniques and radiographic protocols.

There are two main deviations from standard-of-care in our study investigations. Firstly, we will perform the same blood draws and disease assessments but at more frequent intervals than standard-of-care (weekly compared to 3-weekly), to investigate temporal variations in tumour marker level. However, we will limit this increased frequency to the first two chemotherapy cycles and a maximum of one additional blood test per subsequent cycles. Secondly, we may measure participants' serum ctDNA at a similar or lower frequency as the above blood draws. ctDNA measurements, if performeed, will be regularly spaced, with ~4 measurements per patient over four cycles. ctDNA is not routinely measured in standard-of-care.

We may perform other blood tests as per the clinical investigator's judgement. The clinical investigator will perform history and physical examination, as well as review of the adverse events prior to the start of every subsequent cycle of chemotherapy, as per local institution standards. We will enrol participants for the entire duration of their treatment with the selected regimens, up to a maximum of 12 months per participant. We will document the above findings for each participant.

### Sample Size

Since this is a pilot clinical trial with no precedent data, we did not perform an upfront formal sample size calculation. We intend to first recruit 10 patients and perform an interim analysis using the data generated from these participants, which will include formal power and statistical sample size calculations. Based on these outcomes, we will consider cohort expansion or an RCT.

### Data Collection, Management, and Analysis

#### Primary Outcome Measure

The primary outcome measure (Table 1) is the percentage of participants in whom we successfully apply a CURATE.AI profile. This is the main outcome which we will use to judge the technical feasibility of a future RCT and corresponds to the first of our primary objectives. A decision on whether we “successfully apply” the CURATE.AI profile requires expert judgement and cannot be made based on a purely numerical process therefore the statistical analyses will be viewed within the broader framework of clinical relevance. An expert panel, comprising physicians and researchers not involved in the trial, will consider the following factors with careful regard for the individual circumstances of each participant: (1) based on clinical experience, judgement and established indicators, the error/variance analyzed with descriptive statistics of numerical performance measures [i.e., mean absolute error, error distribution and bias (the frequency and extent of under- and overpredicting)] is acceptable to guide clinical decisions; (2) profile can be generated sufficiently early for the participant to potentially benefit; (3) dose-dependent relationship is observed; (4) profile is actionable (i.e., fulfils the clinical investigator's pre-specified safety requirements); (5) systemic changes in the participant which require profile recalibration are rare or readily assimilated into the CURATE.AI algorithm. This is the main outcome which we will use to judge the technical feasibility of the RCT. As per the CONSORT extension statement for pilot clinical trials, we are using “the traffic light system” to define progression criteria for the RCT (33).

#### Secondary and Exploratory Outcome Measures

The secondary outcome measures (Table 1) focus on both technical and logistical feasibility, which constitute our primary objectives. Secondary outcomes include: (1) patient adherence to the prescribed dose; (2) timeliness of CURATE.AI recommendations to the physician in time for the next chemotherapy cycle, across all participants and cycles; (3) physician adherence to CURATE.AI recommended doses; (4) clinically significant dose changes where a participant's CURATE.AI-guided cumulative dose is ≥10% different from the projected standard-of-care cumulative dose.

Exploratory outcome measures (Table 1) mainly relate to efficacy and toxicity, which constitute our secondary objectives. Exploratory outcomes relating to efficacy include: (1) percentage of trial participants with clinical progressive disease defined as the clinical investigator deeming that the patient will not benefit any further from the chemotherapy regimen and considering stopping it, at the time of the first radiological assessment performed as per standard-of-care; as well as (2) temporal variation and (3) maximal reduction in tumour marker level from trial initiation to conclusion. We will also measure clinically relevant toxicities of grades 3–4 as an exploratory outcome, using the Common Terminology Criteria for Adverse Events (CTCAE) version 4.0. Other exploratory outcomes include data collection and exploratory analysis of CEA, CA19-9 and/or other traditional tumour markers in higher frequency serial measurements after modulated dosing in relation to standard frequency readings and other efficacy measures, e.g., RECIST criteria. Finally, we will perform data collection and exploratory analysis of ctDNA as a tumour marker in serial measurements at given clinical context and after modulated dosing, as well as a potential input for CURATE.AI. However, we will not use the analysis of ctDNA to prospectively guide drug dosing in this pilot clinical trial.

#### Statistical Analysis

We will perform and report descriptive statistics of the outcome measures. We will also perform graphical analyses of the temporal variations in tumour marker level. We will not statistically analyse efficacy and toxicity exploratory outcomes.

#### Data Availability

The data generated and/or analysed during the current study are available from the corresponding author on reasonable request.

#### Safety Monitoring and Data Storage

Included in Supplementary Text.

### Patient and Public Involvement

This pilot trial protocol was designed without patient involvement. We did not involve patients in our study design, protocol writing, development of patient-relevant outcomes, nor dissemination of study results. However, we intend to involve patients and the public in the design of the future RCT.

## Discussion

The inclusive recruitment criteria for this pilot clinical trial are enabled by CURATE.AI's personalisation of the treatment and permit substantial variability in the participant population that also reflect the true patient heterogeneity faced in clinical practice. This allows broader applicability of our findings and may also allow us to suggest specific subsets of patients, diseases or chemotherapy regimens where CURATE.AI-guided dosing is particularly beneficial.

Our pilot trial design has several limitations. First, this is a non-randomised pilot clinical trial, which does not simulate an RCT as closely as a randomised pilot. Therefore, it cannot inform us on feasibility issues that may arise from the logistical process or patient acceptability of randomisation, though we view such issues as unlikely in this context. Second, our recruitment criteria intentionally permit substantial variability in the participant population. This is enabled by CURATE.AI's personalisation of the treatment and reflects the true patient population heterogeneity faced in clinical practice and may allow us to identify the factors predicting successful CURATE.AI-guided dosing, for implementation in a future RCT. We nonetheless acknowledge that any identified factors will be based mainly on clinical judgement rather than statistically powered analyses. Third, participants included in this pilot must have raised levels of traditional tumour markers (e.g., CEA or CA19-9). Therefore, our experience with ctDNA in this pilot trial may not be directly generalisable to the intended patient profile in the future RCT if ctDNA were to use as an alternative biomarker for patients who lack elevated levels of traditional tumour markers. Fourth, our primary outcome measure on the successful application of CURATE.AI profiles cannot be measured based only on a numerical process and thus relies on the judgement of an expert panel with consideration for each patient's circumstances. This is inherently subjective. We have nonetheless listed the guiding criteria for the expert panel to reduce any potential bias to the minimum.

## Conclusions

This protocol describes the design of the PRECISE CURATE.AI pilot clinical trial. To date, drug dosing in oncology lacks personalisation. CURATE.AI opens the possibility of personalised dosing for single- and multi-drug regimens, that is dynamically optimized throughout treatment. Furthermore, it is based on a small data set collected only from the treated individual rather than population data. CURATE.AI may thus overcome the challenges that impede the adoption of big data approaches for personalised drug dosing. This pilot will provide technical and logistical feasibility data, as well as exploratory efficacy and toxicity data for sample size calculations, thus laying the clinical foundation for a future RCT of personalised dosing for precision oncology using CURATE.AI.

## Ethics Statement

The studies involving human participants were reviewed and approved by the National Healthcare Group (NHG) Domain Specific Review Board (DSRB), reference: 2020/00334. The patients/participants provided their written informed consent to participate in this study.

## Author Contributions

RS, DH, TK, and ABl: study conception. BT, CT, ABa, DH, RS, TK, and ABl: study design. BT, CT, XT, SP, and ABl: manuscript drafting (introduction). BT, CT, RS, XT, TK, and ABl: manuscript drafting (interventions). BT, CT, RS, TK, and ABl: manuscript drafting (outcomes). BT, CT, HS, SD, VW, RS, TK, and ABl: manuscript drafting (other methods). RS: principal investigator. All authors: critical revision.

## Conflict of Interest

DH, ABl, and TK are inventors of pending and issued patents pertaining to artificial intelligence-based drug development and personalised medicine. DH and TK are shareholders of KYAN Therapeutics, which has licensed intellectual property pertaining to AI-based drug development. The remaining authors declare that the research was conducted in the absence of any commercial or financial relationships that could be construed as a potential conflict of interest.
