# Supplementary material for: Personalised, Rational, Efficacy-Driven Cancer Drug Dosing via an Artificial Intelligence SystEm (PRECISE): A Protocol for the PRECISE CURATE.AI Pilot Clinical Trial
Source: Front Digit Health. 2021 Apr 12;3:635524. doi: 10.3389/fdgth.2021.635524 (PMC8521832; doi:10.3389/fdgth.2021.635524)
Supplement: Supplementary file 1 [file Data_Sheet_1.docx]

# Supplement 1

| Supplementary Material WITHDRAWAL CRITERIA |
| --- |
| A subject may be withdrawn from the study for any of the following reasons:   - - Lost to follow-up   - Withdrawal by subject   - Disease progression   - Toxicity   - Death   - Other   The consequence of study withdrawal is that no new information will be collected from the withdrawn subject and added to the existing data or any database; however, every effort will be made to follow up all subjects for safety. |
| SAFETY monitoring and reporting |
| Definitions |
| **Adverse Event**  An adverse event is defined as any untoward medical occurrence in a participant administered a pharmaceutical product and which does not necessarily have a causal relationship with the treatment. An adverse event can therefore be any unfavourable and unintended sign (including an abnormal laboratory finding), symptom, or disease temporally associated with the use of a medicinal (investigational) product, whether or not the event is considered causally related to the use of the product. Such an event can result from use of the drug as stipulated in the protocol or labelling, as well as from accidental or intentional overdose, drug abuse, or drug withdrawal. Any worsening of a pre-existing condition or illness is considered an adverse event (with the exception of cancer). Clinical signs and symptoms of disease progression are considered adverse events. Laboratory abnormalities and changes in vital signs are considered to be adverse events only if they result in discontinuation from the study, necessitate therapeutic medical intervention, meet protocol specific criteria and/or if the investigator considers them to be adverse events.  **CTCAE term (adverse event description) and grade:** The descriptions and grading scales found in the revised NCI Common Terminology Criteria for Adverse Events (CTCAE) version 4.0 will be utilised for adverse event reporting. A copy of the CTCAE version 4.0 can be downloaded from the CTEP web site (http://ctep.cancer.gov/reporting/ctc.html).    • **“Expectedness”**: Adverse events can be “Expected” or unexpected.  • **Attribution** of the adverse event:  - Definite – The adverse event *is clearly related* to the study treatment.  - Probable – The adverse event *is likely related* to the study treatment.  - Possible – The adverse event *may be related* to the study treatment.  - Unlikely – The adverse event *is doubtfully related* to the study treatment.  - Unrelated – The adverse event *is clearly NOT related* to the study treatment.  **Serious Adverse Event**  A serious adverse event or serious adverse drug reaction is any untoward medical occurrence at any dose that meets any of the following criteria, whether related to study drug or not:  **Death of Participant** An event that results in the death of a participant.  **Life-Threatening** An event that, in the opinion of the investigator, would have resulted in immediate fatality if medical intervention had not been taken. This does not include an event that would have been fatal if it had occurred in a more severe form.  **Hospitalization** An event that results in an admission to the hospital for any length of time. This does not include an emergency room visit or admission to an out-patient facility.  **Prolongation of Hospitalization** An event that occurs while the study participant is hospitalised and prolongs the participant's hospital stay.  **Congenital Anomaly** An anomaly detected at or after birth, or any anomaly that results in fetal loss.  **Persistent or Significant Disability/Incapacity** An event that results in a condition that substantially interferes with the activities of daily living of a study participant. Disability is not intended to include experiences of relatively minor medical significance such as headache, nausea, vomiting, diarrhoea, influenza, and accidental trauma.  **Important Medical Event Requiring Medical or Surgical Intervention to Prevent Serious Outcome** An important medical event that may not be immediately life-threatening or result in death or hospitalization, but based on medical judgment may jeopardise the participant and may require medical or surgical intervention to prevent any of the outcomes listed above (*i.e.,* death of participant, life-threatening, hospitalization, prolongation of hospitalization, congenital anomaly, or persistent or significant disability/incapacity). Examples of such events include allergic bronchospasm requiring intensive treatment in an emergency room or at home, blood dyscrasias or convulsions that do not result in inpatient hospitalization, or the development of drug dependency or drug abuse.  **Spontaneous Abortion** Miscarriage experienced by study participant.  **Elective Abortion** Elective abortion performed on study participant. |
| Collecting, Recording and Reporting of “Unanticipated Problems Involving Risk to Participants or Others” – UPIRTSO events to the NHG Domain Specific Review Boards (DSRB) |
| **UPIRTSO events** refers to problems, in general, to include any incident, experience, or outcome (including adverse events) that meets ALL of the following criteria:   1. **Unexpected**   In terms of nature, severity or frequency of the problem as described in the study documentation (eg: Protocol, Consent documents etc).   1. **Related or possibly related to participation in the research**   Possibly related means there is a reasonable possibility that the problem may have been caused by the procedures involved in the research; and   1. **Risk of harm**   Suggests that the research places participants or others at a greater risk of harm (including physical, psychological, economic, or social harm) than was previously known or recognised.  **Reporting Timeline for UPIRTSO Events to the NHG DSRB.**   1. Urgent Reporting: All problems involving local deaths, whether related or not, should be reported immediately – within 24 hours after first knowledge by the NHG investigator. 2. Expedited Reporting: All other problems must be reported as soon as possible but not later than 7 calendar days after first knowledge by the NHG investigator. |
| Collecting, Recording and Reporting of Serious Adverse Events (SAEs) to the Health Science Authority (HSA) |
| 1. **For Industry sponsored Trials**   All SAEs will be reported to HSA according to the HSA Guidance for Industry “Safety Reporting Requirements for Clinical Drug Trials.”   1. **For Principal Investigator initiated Trials**   All SAEs that are unexpected and related to the study drug must be reported to HSA.  “A serious adverse event or serious adverse drug reaction is any untoward medical occurrence at any dose that:   - Results in death. - Is life-threatening (immediate risk of death). - Requires inpatient hospitalization or prolongation of existing hospitalization. - Results in persistent or significant disability/incapacity. - Results in congenital anomaly/birth defect. - Is a Medically important event.   Medical and scientific judgment should be exercised in determining whether an event is an important medical event. An important medical event may not be immediately life threatening and/or result in death or hospitalization. However, if it is determined that the event may jeopardise the participant and/or may require intervention to prevent one of the other adverse event outcomes, the important medical event should be reported as serious.”  All SAEs that are unexpected and related to the study drug will be reported. The investigator is responsible for informing HSA no later than 15 calendar days after first knowledge that the case qualifies for expedited reporting. Follow-information will be actively sought and submitted as it becomes available. For fatal or life-threatening cases, HSA will be notified as soon as possible but no later than 7 calendar days after first knowledge that a case qualifies, followed by a complete report within 8 additional calendar days. |
| Safety Monitoring Plan |
| The principal investigator (PI) is responsible for appropriate medical care of participants during the study. Investigator will review all participants before each treatment cycle for monitoring of toxicity. If any participant reports an adverse event, the investigator should follow the participant until the event is either resolved or assessed as stable.  For the prospective study, the medical team will strip the data of any unnecessary personal data information and code it per participant. The code key will be kept in a standalone computer. The coded data will be provided to the data analysis team members the same day as receiving the readout results by the medical team.  For the exploratory analysis of ctDNA suitability, the medical team will enlist a staff member to act as a trusted third party and de-identify the data. The aggregated, de-identified data will be provided to the data analysis team members to prevent the re-identification of the participants. |
| Complaint Handling |
| Patients will be advised as per the informed consent form that they may contact the Principal Investigator or DSRB secretariat if they have any complaints. |
|  |
| DATA STORAGE and security |
| Data Quality Assurance |
| Data integrity will be assured by matching and verifying with the data source. The PI will be subjected to HSA and DSRB audits when needed. |
| Data Entry and Storage |
| A REDCap (Research Electronic Data Capture) database will be established specifically to collect the data for the registry. Each patient recruited into the study will be assigned a unique patient number (UPN), and the patient’s biosamples will be labelled using the UPN, with no direct reference to the patient's other identifying information. Information from the source documents will be transcribed onto an electronic database that is password protected in a user designated and password protected computer in the Department. Personnel in the laboratory have no direct access to the clinical history database or other patient information. Information pertaining to the patient that arises from the research will not become part of the patient's medical record. All records will be kept for a minimum period of 6 years following the date of study closure according to ICH GCP guidelines, or longer as applicable per institution guidelines. |

| Retention of Trial Documents |
| --- |
| Source Documents  Original documents, data, and records (e.g. medical records, raw data collections forms, pharmacy dispensing records, recorded data from automated instruments, laboratory data) that are relevant to the clinical study will be adequately prepared and maintained. These documents are designed to record all observations and other pertinent data for each participant enrolled in this clinical study. Source records will adequately reconstruct all data entered into the case report forms, which will be completed in English.  Archival of Records  The investigators will retain records required to be maintained under this part for a period of 15 years following the completion or discontinuation of the study. The investigators will retain protocols, amendments, IRB approvals, copies of the signed and dates consent forms, medical records, case report forms, drug accountability records, all correspondence, and any other documents pertaining to the conduct of the study. |
|  |
| ETHICAL CONSIDERATIONS |
| Informed Consent |
| No investigator may involve a human being in research unless the investigator has obtained the legally effective informed consent of the patient or the patient’s legally authorised representative. An investigator shall seek such consent only under circumstances that provide the prospective patient or the patient’s legally authorised representative sufficient opportunity to consider whether or not to participate, and minimise the possibility of coercion or undue influence. The information that is given to the patient or the representative shall be in a language understandable to the patient or representative.  Before implementing any study procedure, informed consent will be documented in the subject case histories and by the use of a written consent form approved by the DSRB and signed and dated by the patient or the patient’s legally authorised representative at the time of consent. A copy of the signed informed consent will be given to the patient or patient’s legally authorised representative. The original, signed consent will be maintained by the investigator and available for inspection by the regulatory authority at any time. In obtaining and documenting informed consent, the investigator will comply with the SGGCP guidelines and the ethical principles that have their origin in the Declaration of Helsinki.  The patient will be informed about the background and aims of the study. The patient will be told of her right to withdraw from the study at any time without any penalty with regards to the continuation of care at this institution and by the same physicians as she chooses. The patient will be told that tissue and blood samples obtained will be assigned unique patient numbers (UPN) to ensure patient confidentiality. |
| Confidentiality of Data and Patient Records |
| Protection and privacy of the personal data of individuals are covered under the Personal Data Protection Act 2012. Patient medical information obtained as part of this study is confidential, and must not be disclosed to third parties, except as noted below. The patient may request in writing that medical information be given to his/her personal physician.  The investigator/institution will permit direct access to source data and document by regulatory authorities. The access may consist of study-related monitoring, audits, DSRB reviews and regulatory authority inspection.  A REDCap (Research Electronic Data Capture) database will be established specifically to collect the data for the registry. Each patient recruited into the study will be assigned a unique patient number (UPN), and the patient’s biosamples will be labeled using the UPN, with no direct reference to the patient's other identifying information. Information from the source documents will be transcribed onto an electronic database that is password protected in a user designated and password protected computer in the Department. Personnel in the laboratory have no direct access to the clinical history database or other patient information. Information pertaining to the patient that arises from the research will not become part of the patient's medical record. All records will be kept for a minimum period of 6 years following the date of study closure according to ICH GCP guidelines, or longer as applicable per institution guidelines.  Information collected includes demographic characteristics, cancer history and pathological information, past and present cancer treatment history of the study subject. The subjects’ progress may be followed up periodically (approximately every 6 months) through the medical records, and subsequent cancer treatments, progression of cancer, and survival outcome will be updated. Important treatment information that may be collected include: the drug regimens, drug doses, intent of treatment, haematologic and non-haematologic toxicities, and hospitalization episodes that may be related to treatment. Subjects may be followed-up till death. Toxicities will be graded using the Common Terminology Criteria for Adverse Events version 4.03 (CTCAE). |
